# Traditional Chinese Medicine Polysaccharides-Based Nano-Drug Delivery Systems: Design Strategies and Combination Platforms in Tumor Therapy

**DOI:** 10.3390/life16050838

**Published:** 2026-05-19

**Authors:** Qianru Chen, Yixuan Gan, Ruiyi Tang, Jianan Zhang, Di Wu

**Affiliations:** Key Laboratory of Neuropharmacology and Translational Medicine of Zhejiang Province, School of Pharmaceutical Sciences and School of Basic Medical Sciences, Zhejiang Chinese Medical University, Hangzhou 310053, China; 18858739615@163.com (Q.C.); ganyx6670@163.com (Y.G.); 18334380115@163.com (R.T.); 202212211501009@zcmu.edu.cn (J.Z.)

**Keywords:** traditional Chinese medicine polysaccharides, tumor, nano drug delivery system, structural modification, combination therapy

## Abstract

Conventional tumor therapies and traditional nanocarrier delivery systems both suffer from multiple limitations. Traditional Chinese medicine polysaccharides (TCMPs), which possess excellent biocompatibility, low toxicity, structurally modifiable characteristics and inherent pharmacological activities, have emerged as promising functional materials for constructing antitumor drug delivery systems and are being gradually applied in tumor combination therapy. This review systematically summarizes the common structural characteristics, biological functions, and structure–activity relationships of representative TCMPs. It further discusses the principal modification strategies used to construct TCMPs-based drug delivery systems and highlights recent progress in their applications in immunotherapy-centered combination therapy. In addition, it outlines their mechanistic basis and therapeutic potential for improving drug delivery efficiency and antitumor efficacy. This review provides theoretical references for the optimal design of such novel systems and guidance for their applications in precision treatment of tumors, while also pointing out some challenges in the translational research of such systems.

## 1. Introduction

Malignant tumors remain a major threat to human health, and currently available therapies are still insufficient to achieve safe and effective cure in many patients [1]. Although surgery, chemotherapy, and other widely used clinical therapies have achieved considerable clinical success, their overall efficacy is still restricted by several major limitations, such as poor selectivity, severe systemic toxicity and inadequate drug accumulation at tumor sites. Therefore, it remains an urgent priority to develop safer and more efficient approaches for tumor treatment.

Currently, nano-drug delivery systems (NDDS) have captured extensive attention as a promising avenue to bypass the limitations of conventional anticancer therapies [2]. By employing carriers including inorganic nano-materials, polymeric nanoparticles, and liposomes, these systems enable controlled drug release and better target therapeutic agents, leading to higher therapeutic efficacy and lower systemic toxicity [3,4]. Despite significant progress in related research, conventional nanocarriers still face challenges such as potential nanotoxicity, low bioavailability, and poor pharmacokinetic properties, with clinical studies indicating that only about 1% of the injected dose reaches the targeted tumor tissue [5,6]. These limitations urge researchers to explore natural biomaterials with better biosafety and functional versatility to construct a new drug delivery platform.

Among numerous natural materials, bioactive components from traditional Chinese medicine (TCM) have aroused widespread interest due to their great biocompatibility, renewability, low toxicity, and profound pharmacological activity. These components can not only exhibit excellent therapeutic effects on their own, but also serve as drug delivery carriers, thereby providing the possibility for synergistic treatment after drug loading [7]. Among them, traditional Chinese medicine polysaccharides (TCMPs), as a class of structurally diverse natural macromolecules, show considerable application potential in nanomedicine. TCMPs are associated with a broad range of biological activities, including immunomodulation, anti-inflammation, antitumor activity, and hypoglycemic effects, and some of them are widely used clinically, such as lentinan (LNT) and *Astragalus* polysaccharides (APS) [8,9,10,11]. In addition, TCMPs possess abundant functional groups represented by hydroxyl, which provide versatile sites for chemical modification and functional design. Their intrinsic hydrophilicity, gel-forming capacity, biodegradability, and favorable biosafety profile further support their use in biomedical applications and NDDS [12,13,14]. Owing to these advantages, TCMPs have been increasingly investigated as promising building blocks for the development of advanced nanocarriers. Emerging studies have demonstrated that TCMPs-based nanocarriers can improve drug bioavailability and tumor-targeting efficiency, enable stimulus-responsive drug release, and modulate the immune microenvironment within tumors [15,16,17,18].

**Figure 1:**
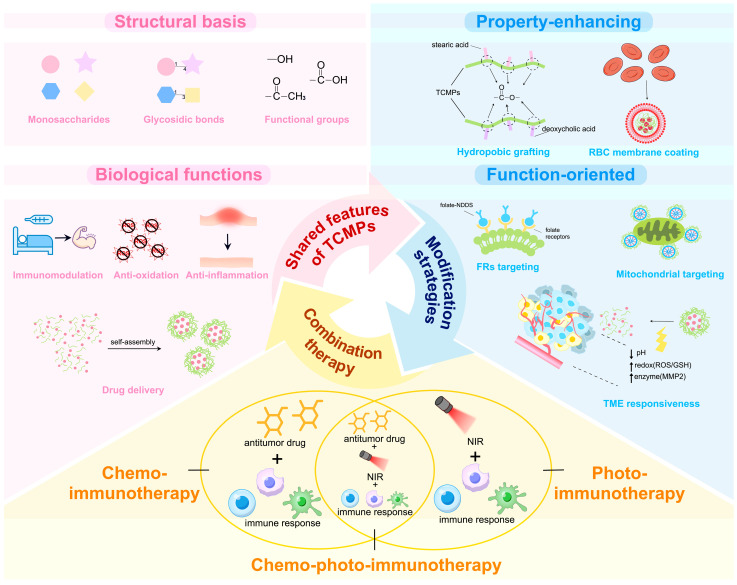
The shared features of TCMPs, modifications of TCMPs-based NDDS, and their applications in immune-centered combination therapy. This schematic summarizes the common structural basis, intrinsic biological functions, and drug-delivery potential of TCMPs and illustrates the principal modification strategies used to optimize TCMPs-based nano-drug delivery systems, including property-enhancing and function-oriented approaches. It also highlights the representative applications of TCMPs-based platforms in chemo-immunotherapy, photo-immunotherapy, and chemo-photo-immunotherapy. Abbreviations: traditional Chinese medicine polysaccharides (TCMPs), red blood cell (RBC), tumor microenvironment (TME), reactive oxygen species (ROS), glutathione (GSH), matrix metalloproteinase 2 (MMP2), near-infrared irradiation (NIR).

To date, TCMPs-based NDDS have been explored in a variety of biomedical fields, including wound healing, anti-inflammatory therapy, and immune therapy [19,20,21]. Although numerous reviews have summarized TCMPs-based drug delivery, systematic reviews specifically focusing on their tumor therapy applications and design strategies remain scarce. Thus, this review will systematically summarize the structural characteristics and biological functions of some representative TCMPs, outline their latest advances in structural design and functionalization strategies of NDDS, and generalize their applications in immunotherapy-centered tumor combination therapy (Figure 1).

## 2. Structural Characteristics and Biological Functions of Traditional Chinese Medicine Polysaccharides

TCMPs are natural polysaccharides sourced from medicinal materials that are used according to TCM theory. Although they originate from different medicinal sources and exhibit substantial structural diversity, TCMPs still share a number of common structural features that determine their biological activities and material properties (Table 1). These common structural characteristics provide the fundamental basis for pharmacological activity, carrier construction, and subsequent functional modification. Therefore, this section focuses on the common structural characteristics of TCMPs, their derived biological and carrier functions, and the structure–function relationships governing their pros and cons in drug delivery.

### 2.1. Common Structural Characteristics of Traditional Chinese Medicine Polysaccharides

TCMPs generally comprise various monosaccharide residues including glucose, galactose, arabinose, rhamnose, and mannose [48,49,50]. The exact monosaccharide composition varies according to extraction process, purification procedure, and analytical method, but the coexistence of multiple monosaccharide residues is one of the common structural features of TCMPs. This compositional diversity gives rise to considerable heterogeneity in physicochemical characteristics [51,52,53]. For example, *Polygonatum sibiricum* polysaccharides prepared by different methods differed considerably in monosaccharide profiles. Alkali-extracted polysaccharides possessed higher molecular weight and no galacturonic acid, thus displaying greater apparent viscosity, stronger gelation, and better rheological properties. By contrast, enzyme- and freeze–thaw-extracted polysaccharides exhibited lower molecular weight and simpler monosaccharide composition, which contributed to higher water solubility, improved dissolution, stronger hydration, and more stable solution behavior [54].

In addition to monosaccharide composition, TCMPs typically possess complex glycosidic linkages. Their monosaccharide residues are linked by various glycosidic bonds, such as (1→3), (1→4), and (1→6) linkages, forming either relatively linear or highly branched chain structures [26,55,56]. The type of glycosidic linkage also determines the stiffness, flexibility, and spatial arrangement of the polysaccharide chains. It is well known that β-linked glycosidic bonds especially β-(1→4) exhibit restricted rotational freedom and thus confer higher chain rigidity, whereas α-linked bonds allow greater conformational flexibility. In addition to the type of glycosidic linkages, the branching condition can also affect the physicochemical and solution properties of polysaccharides. An increase in degree of branching (DB) weakens intermolecular interactions and enhances the hydrophilic association between polysaccharide chains and water molecules, thereby improving water solubility [57]. For instance, amylose exhibits much lower solubility in cold water than amylopectin. Meanwhile, a higher DB leads to reduced solution viscosity and improved hydrodynamic behavior. A highly branched β-glucan from *Ganoderma lucidum* spores demonstrates excellent water solubility and low viscosity, which serves as a typical example [58].

Another important common feature of TCMPs is the abundance of functional groups. TCMPs typically contain numerous hydroxyl groups, and acidic TCMPs usually possess carboxyl groups [59]. These groups not only contribute to the strong hydrophilicity of TCMPs, but also provide versatile chemical sites for esterification, Schiff base formation, grafting, crosslinking, and ligand conjugation. Thus, the rich functional-group distribution of TCMPs constitutes the direct chemical basis for their structural tunability and for the development of functionalized NDDS.

Taken together, TCMPs share several common structural characteristics, including diverse monosaccharide composition, variable glycosidic bonds, and abundant reactive groups. These common features do not merely define their chemical identities but also provide the material foundation.

### 2.2. Common Pharmacological Activities and Carrier-Related Functions of Traditional Chinese Medicine Polysaccharides

A multitude of studies have revealed that most TCMPs have the capabilities of immunomodulation, anti-oxidation, and anti-inflammation, all of which are crucial in cancer treatment [32,60,61,62,63,64,65]. Therefore, the antitumor effects of TCMPs are generally mediated through these activities, among which immunomodulation appears to be particularly important in tumor therapy [66,67]. For instance, LNT, a typical representative with pronounced immune-enhancing effects, is a classic immunological adjuvant. These properties distinguish TCMPs from conventional inert carriers and represent one of their most important advantages in the field of tumor therapy.

In addition to their pharmacological activities, TCMPs have been confirmed to possess excellent carrier functions, and a variety of TCMPs-based NDDS have been developed based on their unique physicochemical properties [68,69,70,71]. Firstly, TCMPs have intrinsic hydrophilicity, which facilitates their dispersion in biological media and provides a favorable support for the construction of delivery matrices such as hydrogels, micelles, nanogels and films [72,73]. Secondly, their molecular surfaces are rich in reactive groups, making them easy for chemical modification [29,70]. The introduction of hydrophobic moieties, targeting ligands, responsive bonds and crosslinking structures can effectively improve their delivery performance. Moreover, in most cases, rationally modified TCMPs can undergo self-assembly, thereby enabling drug encapsulation, sustained release and controlled delivery.

Remarkably, TCMPs combine carrier functions with pharmacological activities. While serving as drug delivery carriers, they can exert their inherent pharmacological effects, especially immunomodulation, which can synergize with the loaded drugs and mitigate the side effects induced by chemotherapeutic agents [17].

### 2.3. Structure–Function Relationships and Limitations of Traditional Chinese Medicine Polysaccharides

The common pharmacological and carrier-related functions of TCMPs are closely associated with their structural features. In other words, the biological performance of TCMPs is governed by the interplay of monosaccharide composition, glycosidic bonds, and functional groups. Understanding these structure–function relationships is essential not only for explaining the endogenous activities of TCMPs, but also for rationally designing TCMPs-based NDDS.

One of the primary structural factors influencing biological activity is monosaccharide composition. Different sugar residues and their relative proportions may affect immune recognition, receptor binding, and cellular interactions. For example, galactose-rich TCMPs, as represented by ASP, may exhibit preferential accumulation in the liver because galactose residues can interact with the asialoglycoprotein receptor (ASGPR) on hepatocytes [27,74]. Likewise, APS show selective uptake in 4T1 breast cancer cells, likely due to interaction between their glucose residues and the highly expressed glucose transporters such as GLUT1. These properties are widely applied in TCMPs-based targeted drug delivery [24,28,75]. The relative ratios of distinct monosaccharides may also play an important role in determining the properties of TCMPs. It was found that mannose–glucose branched polysaccharides with an approximate ratio of 2.4 to 1 possess greater conformational flexibility, allowing tighter folding and better encapsulation of drug molecules [26].

Glycosidic linkage type also plays key roles in determining the biological effects of TCMPs. In terms of pharmacological effects, specific glycosidic bonds serve as the structural basis for TCMPs to exert biological activities. Polysaccharides containing β linkages, especially β-(1→3) linkage, generally exhibit strong immune-stimulating and antitumor potential, and the triple-helical structure based on β-glycosidic linkages is an essential basis for the antitumor efficacy of LNT and GLP [76,77,78,79]. Linked via β-(1→3) and β-(1→6) bonds, LNTs tend to form a triple-helix conformation. This structure specifically recognizes the Dectin-1 receptor of immune cells and activates downstream Dectin-1/Syk/NF-κB signaling pathways, thus enhancing immunomodulatory and antitumor activities [39,80]. The glycosidic linkage type may affect the stability of TCMPs as drug carriers. Polysaccharides mainly containing β-(1→4) bonds are usually more stable than polysaccharides mainly containing α-(1→4) bonds because the latter are typically hydrolyzed by α-amylase. To conclude, glycosidic bonds are indispensable to both the exertion of pharmacological activities and the realization of carrier functions, thereby representing the core for understanding the structure–function relationship of TCMPs.

Functional groups provide another important link between structure and function. Hydroxyl, carboxyl, and acetyl are directly involved in hydrogen bonding, electrostatic interactions, receptor binding, and redox-related chemistry, which may affect both pharmacological activity and material behavior. A higher proportion of hydroxyl groups promotes the elimination of reactive oxygen species (ROS) and amplifies the antioxidant property of TCMPs. In addition, functional groups provide abundant reactive sites for chemical modification, thereby enabling the elevation of biological effects of TCMPs and their transformation into structurally optimized delivery carriers [81]. Therefore, the rich functional-group distribution of TCMPs serves as the structural basis for constructing functionalized NDDS.

However, while TCMPs exhibit diverse pharmacological activities and carrier-related functions, they still suffer from inherent limitations. First, the structural heterogeneity of TCMPs hinders the establishment of precise and universal structure–function relationships. This complexity reduces reproducibility and complicates quality control. Second, although abundant hydrophilic groups contribute to water compatibility and biosafety, they also often result in insufficient hydrophobic drug-loading capacity and weak intrinsic self-assembly ability. Third, the flexible molecular chains and naturally occurring non-covalent assemblies of many TCMPs may lead to inadequate mechanical strength or insufficient stability in vivo. Fourth, most TCMPs still fail to meet the demands of complex tumor treatments; thus, they are unable to achieve precise tumor delivery, efficient intracellular transport, or robust stimulus responsiveness.

To conclude, the native structures of TCMPs provide the basis for their intrinsic biological activity and carrier construction, but they also impose limitations on drug loading, stability, and targeting. Hence, these challenges necessitate the modification of TCMPs to enhance the development of TCMPs-based NDDS.

## 3. Modifications of Traditional Chinese Medicine Polysaccharides-Based Drug Delivery Systems

**Figure 2:**
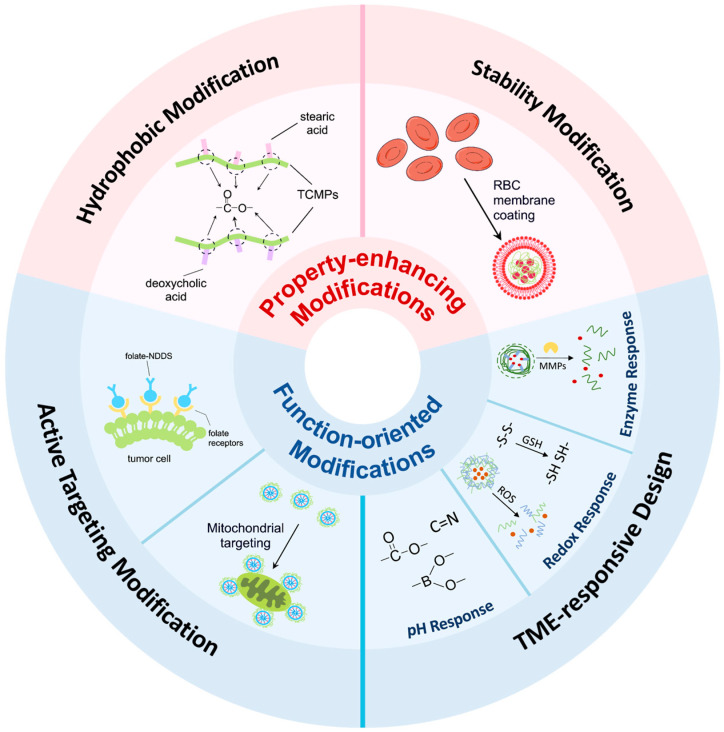
Property-enhancing and functional-oriented modifications of TCMPs-based NDDS. This schematic summarizes the principal modification strategies used to optimize TCMPs-based nano-drug delivery systems. Property-enhancing strategies mainly include hydrophobic modification and stability modification, such as stearic acid or deoxycholic acid grafting, and RBC membrane coating, which improve self-assembly, drug loading, structural integrity, and in vivo persistence. Function-oriented strategies mainly include targeting modification and tumor microenvironment-responsive design, such as folate receptor targeting, mitochondrial targeting, and pH-, redox-, or enzyme-responsive regulation, which enhance tumor-selective accumulation and controlled drug release.

The modification of TCMPs represents a central strategy for overcoming intrinsic limitations and improving the delivery efficiency of TCMPs-based drug delivery systems. Modification strategies can be broadly classified into property-enhancing and functional-oriented approaches (Figure 2). The former focuses on improving drug loading and stability, such as introducing hydrophobic moieties to increase hydrophobicity, enabling self-assembly into micelles suitable for encapsulating hydrophobic drugs. Membrane-coating techniques can further prolong systemic circulation and enhance carrier stability in vivo. The latter emphasizes precise tumor delivery, including targeting of tumor microenvironment (TME)-responsive designs which exploit the distinctive physiological characteristics of tumor tissues, allowing controlled on-site release. These strategies enhance drug loading, stability, accumulation within tumors, and reduce off-target toxicity, while also enabling synergistic effects with chemotherapy or immunotherapy. They collectively establish a foundation for TCMPs-based NDDS in tailored cancer treatments.

### 3.1. Property-Enhancing Modification

#### 3.1.1. Hydrophobic Modification

Many TCMPs are water-soluble and therefore exhibit a limited capacity to encapsulate antitumor drugs, most of which are lipophilic. Consequently, hydrophobic modification is required to enable the loading of lipophilic drugs and achieve effective drug delivery.

Among various hydrophobic modification strategies, stearic acid (SA) and deoxycholic acid (DOCA) grafting are the most commonly used approaches for constructing amphiphilic TCMPs-based nanocarriers. SA is a simple and widely used hydrophobic moiety that mainly improves amphiphilicity and hydrophobic drug loading, whereas DOCA with a rigid steroidal structure and two hydroxyl groups may provide additional modification sites. Both SA and DOCA contain a carboxyl group and can be grafted onto polysaccharides through esterification, thereby achieving hydrophobic modification. After grafting hydrophobic moieties, TCMPs become amphiphilic and can self-assemble into core–shell structures. It is worth noting that the degree of substitution (DS) of hydrophobic molecules may affect the performance of the self-assembled micelles. Zhang et al. reported that within a certain range, a higher degree of substitution resulted in a lower critical aggregation concentration (CAC), favoring micelle formation with a smaller polydispersity index (PDI) [82]. This was because a greater number of hydrophobic segments were introduced, leading to a higher tendency to form a hydrophobic core, thereby lowering the concentration required for micellization and enhancing the stability of the formed micelles. However, excessively high DS will result in overly strong hydrophobicity, thereby impeding micelle formation via self-assembly. This core–shell structure can more effectively encapsulate hydrophobic drugs, thereby prolonging circulation and enhancing bioavailability [83]. For instance, SA-modified BSP self-assembled into polymeric micelles in aqueous media, and when loaded with docetaxel (DTX), it significantly prolonged the release profile and exhibited more pronounced tumor cell inhibitory activity compared to free DTX [15]. Pharmacokinetic parameter analysis indicated that SA-BSP delayed DTX elimination while maintaining sustained plasma concentrations [15]. Furthermore, DOCA-modified ASP loaded with doxorubicin (DOX) realized sustained drug release and exhibited more potent anti-proliferation effect on HepG2 cells compared to free DOX [27].

Collectively, hydrophobic modification of TCMPs typically employs esterification reactions as the reaction mechanism. This modification significantly enhances the delivery efficiency of hydrophobic antitumor drugs.

#### 3.1.2. Stability Modification

When used as drug delivery carriers, TCMPs often face the critical bottleneck of insufficient stability. Some TCMPs suffer from poor in vivo stability, and they may rapidly be recognized and cleared by the mononuclear phagocyte system in organs, particularly the liver and spleen, thereby shortening circulation time and impairing effective delivery. Therefore, enhancing structural stability and delaying in vivo clearance have become core prerequisites for advancing TCMPs delivery systems toward effective clinical application.

Bio-membrane coating offers a distinct stabilization approach by shielding carriers from immune recognition and prolonging the circulation of NDDS. Curcumin (Cur) is a natural bioactive compound with notable antitumor activity [84]. An APS-based system coated with red blood cell membrane (RBCm) containing Cur demonstrated higher liver-targeting ability after 24 h and produced stronger anti-hepatoma efficiency than the uncoated system, highlighting the potential of biomimetic encapsulation to enhance delivery persistence and therapeutic performance [23].

However, exploration of stability-enhancing modifications for TCMPs-based NDDS remains insufficient. Beyond RBCm, a variety of biological membranes, including macrophage membranes, cancer cell membranes, platelet membranes, and exosome-derived membranes, have demonstrated the ability to prolong systemic circulation, reduce opsonization, and improve immune evasion of nanocarriers [85]. Among them, macrophage membrane coating may be particularly promising for TCMPs-based NDDS [86]. Since most TCMPs possess intrinsic immunomodulatory activities, the integration of macrophage membrane camouflage could not only enhance colloidal stability and blood circulation time, but also synergistically regulate the tumor immune microenvironment through inflammation-homing and immune-interactive properties, thereby improving the efficacy of tumor immunotherapy.

In addition to bio-membrane coating, numerous alternative strategies can also be employed to improve the stability of TCMPs-based NDDS. For example, Gao et al. and Xiang et al. combined protein components with natural polysaccharides and formed stable nanoplatforms, which effectively reduced premature drug leakage and significantly promoted the stability of drugs [87,88]. Such findings suggest that rational structural engineering can substantially improve the physicochemical stability and in vivo performance of TCMPs-based NDDS. Future studies should therefore move beyond single stabilization paradigms and systematically explore various modification strategies.

In conclusion, stabilization strategies enable TCMPs-based carriers to exhibit sustained and reliable performance in vivo, transforming their inherently rapid clearance into controlled and prolonged drug exposure. Continued improvements in stability-oriented engineering will further strengthen the therapeutic potential of TCMPs delivery systems in tumor treatment.

### 3.2. Functional-Oriented Modification

#### 3.2.1. Targeting Modification

Other than the liver-targeting property of ASP and that APS is preferentially internalized by 4T1 cells, most TCMPs exhibit limited or no intrinsic targeting ability. Consequently, additional targeting modification is often required to achieve more efficient and precise drug delivery to tumor tissues. Tumor cells differ from normal cells in several aspects, including the overexpression of specific surface receptors and mitochondrial alterations. These characteristics provide targets for functional modification of TCMPs-based NDDS, allowing them to actively recognize tumor cells and improve therapeutic accuracy.

Folate (FA) is essential for cellular growth, and rapidly proliferating tumor cells exhibit heightened demand for FA. Consequently, many tumor cells overexpress folate receptors (FRs) to facilitate the uptake of FA [89]. After grafting onto the hydroxyl groups of TCMPs, FA confers FRs-targeting capability to TCMPs by exploiting the high affinity between FA and FRs, thus accelerating tumor cell uptake of the delivery system. It was found that 4T1 cells and SKOV-3 cells showed uptake preference of FA-modified TCMPs-based NDDS than non-targeted NDDS. In vivo imaging further demonstrated that FA-modified NDDS accumulated preferentially at tumor sites compared with free drugs and non-targeted micelles, while exhibiting minimal distribution in normal organs [16,90]. These experimental results indicate that FA modification can significantly enhance the targeting efficiency of antitumor drugs while reducing their toxic effects on normal cells. Although numerous studies successfully applied FA to develop new delivery platforms with FRs’ targeting ability, there still remains a problem that hinders the clinical applications of these NDDS. The FRs expressed in normal epithelial tissues, such as kidneys, may disrupt the targeting procedure of the FA-modified NDDS and lead to off-target accumulation and toxicity. Therefore, it is urgently necessary to explore and develop more efficient and safer active targeting strategies for precise drug delivery.

In recent years, mitochondria-targeting tumor therapy has attracted more attention [91,92]. Mitochondria in tumor cells display aberrant characteristics such as elevated membrane potential and metabolic abnormalities compared to normal cells [93,94]. An increase in membrane potential can markedly promote the accumulation of positively charged substances within mitochondria [95]. Therefore, introducing cationic-targeting groups can confer mitochondrial targeting capability to TCMPs-based nanocarriers through electrostatic interactions, thereby reducing mitochondrial membrane potential and activating the mitochondrial apoptotic pathway to achieve antitumor therapeutic effect. Compared to the FA-grafting method, the off-targeting risk of mitochondria-targeting therapy is lower, but the introduction of the positively charged substances may trigger some side effects including hemolysis, protein corona formation, and rapid systemic clearance. Accordingly, there is an urgent need to take action to avoid such side effects. Because some of the mitochondria-targeting molecules such as berberine (BBR) are lipophilic cations, it is desirable to cover these lipophilic cations with hydrophilic anionic groups. Notably, ASP are naturally anionic polysaccharides with liver-targeting ability; thus, they can protect the cations and facilitate the internalization of NDDS into hepatocytes via ASGPR. Researchers commonly utilize TME-responsive functional groups to covalently link ASP with mitochondrial-targeting moieties to construct amphiphilic conjugates [29,96]. Upon self-assembly into micelles, the hydrophobic segments are masked by the outer hydrophilic ASP layer. After ASP specifically mediates the efficient internalization of NDDS into hepatocytes, the intracellular TME stimulus triggers the cleavage of responsive linkages, thereby disassembling the micellar structure. Subsequently, the cationic mitochondrial-targeting segments are fully exposed, which can efficiently target the mitochondria of hepatocellular carcinoma cells and achieve precise drug release. This rational design fully takes advantage of the inherent properties of ASP, providing a feasible and promising strategy for the construction of mitochondria-targeted TCMPs-based intelligent NDDS.

Targeting modification is not merely an additional decoration for TCMPs-based NDDS, but a key strategy for transforming these systems from general drug carriers into precision delivery platforms. Folate-mediated targeting mainly addresses insufficient tumor cell recognition, whereas mitochondria-targeting modification further overcomes the limitation that many carriers still fail to reach apoptosis-related subcellular sites. In addition, TCMPs with partial intrinsic targeting properties such as ASP may further broaden their delivery selectivity after additional targeting modification, thereby enhancing the overall functionality of NDDS. These findings suggest that the real significance of targeting design lies not simply in stronger uptake but in more precise spatial control over where drugs act at the tissue, cellular, and subcellular levels. Such strategies support a more rational design of tumor-feature-matched nanoplatforms.

#### 3.2.2. Tumor Microenvironment-Responsive Design

**Figure 3:**
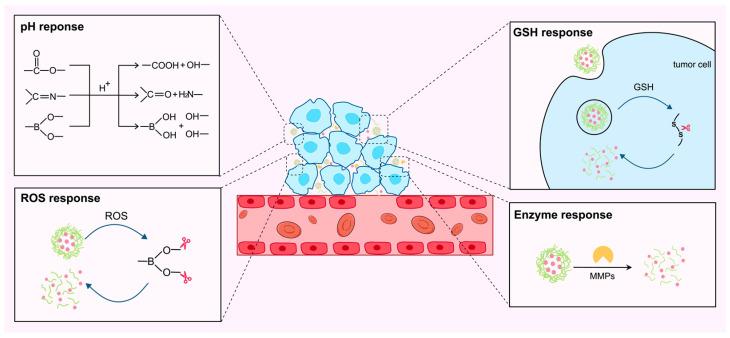
Mechanisms of action of TME-responsive systems. The TME-responsive strategies include pH response, redox response, and enzyme response. The pH response mainly relies on the hydrolysis of ester bonds, Schiff base bonds, and borate ester bonds in an acidic environment. The redox response includes both ROS-responsive and GSH-responsive mechanisms: the former arises from the cleavage of structures such as borate ester bonds under high ROS levels, while the latter arises from the cleavage of structures such as disulfide bonds under high GSH levels. The enzyme response is primarily achieved by introducing peptide that can be specifically cleaved by enzyme such as MMPs. Once these responses are triggered, the TCMPs-based NDDS disassemble and release the drug, thereby achieving precise targeting.

TME-responsive design has become one of the most widely applied strategies in drug delivery systems, as it enables tumor-targeted delivery and controlled drug release [97]. The mildly acidic pH, abnormal redox conditions, and overexpression of certain enzymes within the TME provide the fundamental basis for such responsive regulation. Accordingly, current TME-responsive design strategies are mainly focused on pH-responsive, redox-responsive, and enzyme-responsive systems (Figure 3).

##### pH Response

Tumor tissues commonly exhibit an acidic microenvironment, which is primarily attributable to the enhanced glycolytic pathways of cancer cells. Even under aerobic conditions, these cells produce substantial amounts of lactic acid, resulting in a sustained extracellular pH of approximately 6.5. This acidic TME provides an ideal triggering condition for developing pH-responsive NDDS. Current strategies to impart acid sensitivity to TCMPs include grafting of fatty acids to introduce acid-sensitive ester bonds and the incorporation of Schiff base or borate bonds, enabling TME-specific drug release. It is worth mentioning that SA or DOCA grafting not only achieves hydrophobic modification but also endows TCMPs with the pH-responsive profile, enabling acid-dependent drug release. It is probably because ester bonds are more readily hydrolyzed in acidic environments, thereby disrupting the structure of the carrier and releasing the drug from the hydrophobic core. Studies showed that the drug release rate of SA-BSP micelles decreases sequentially at pH 5.0, 6.0, and 7.4 [33]. Similarly, DOX-loaded ASP-DOCA nanoparticles showed cumulative release rates of only 31% and 39% at pH 7.4 and pH 6.0, respectively, with a marked increase to 56% at pH 5.0 [27]. Therefore, drugs are more readily released in the acidic TME when the skeleton of NDDS contains ester bonds.

Schiff base formation is a condensation reaction that generates a C=N bond between carbonyl and amino groups. Schiff base bonds undergo hydrolysis in acidic environments, thus rendering them highly promising for the design of responsive NDDS. Schiff base-based pH-responsive modification is generally achieved by first converting hydroxyl-rich TCMPs into aldehyde-containing derivatives, followed by condensation with amino-containing drugs to form acid-labile C=N linkages. In this strategy, the abundant hydroxyl groups on TCMP chains provide the structural basis for oxidation and subsequent conjugation, while the Schiff base bonds undergo responsive cleavage in the acidic TME, thereby promoting the selective drug release of the NDDS in tumor regions. This modification mainly addresses the limitation of insufficient tumor-specific release in conventional NDDS, thus amplifying the therapeutic efficiency of chemotherapeutics. For instance, DOX-conjugated oxidized LNT showed only 13.6% cumulative release at pH 7.4 after 48 h, whereas the value increased to 42.3% at pH 6.5 and 63.1% at pH 5.0 [17]. A similar trend was also observed in the LBP system, in which only about 10% of DOX was released at pH 7.4 after 72 h, but the release rate increased to about 53% at pH 5.0 [45].

Borate ester-based responsive modification is generally achieved through the reversible reaction between hydroxyl groups on TCMPs and boronic acid groups, thereby introducing dynamic linkages into the carrier structure. In this strategy, the abundant hydroxyl groups of TCMPs provide the structural basis for borate ester formation, while the resulting bonds can undergo cleavage under acidic conditions of TME and thus promote stimulus-responsive drug release. Moreover, TCMPs themselves act as biologically active carriers with antitumor effect and may cooperate with loaded drugs to enhance tumor therapy. For example, borate ester linkages have been used to construct both pH-responsive core–shell systems and pH/ROS dual-responsive nanogels based on APS. These systems showed accelerated drug release under acidic conditions, and nanogel BAI@ASPOBA loaded with baicalein (BAI) further demonstrated that the antitumor effect resulted from the combined action of BAI and the intrinsic activity of APS [30,96].

Although ester bonds, Schiff base bonds, and borate bonds can all utilize the acidic nature of TME to achieve drug release, their key points are not entirely the same. Modifications involving ester bonds primarily manifest as the acquisition of acid-promoted release properties as a secondary benefit, in addition to hydrophobic construction and enhanced drug-loading capacity. Furthermore, their acid responsiveness is less sensitive than that of Schiff base and borate bonds; therefore, their advantage lies in balancing material assembly with drug release control. Schiff base bonds are more suitable for reversible coupling between drugs and polysaccharide scaffolds. They can maintain relative stability under physiological conditions while achieving significant cleavage and drug release in acidic environments, making them more representative in enhancing selective release at the tumor site. Borate bonds are dynamic reversible bonds that not only confer acid-sensitive dissociation capabilities to the system but also often allow for the integration of multiple responses, making them highly scalable for the construction of more complex smart delivery systems. Consequently, pH-responsive design should place greater emphasis on matching the choice of bond type with the inherent characteristics of the TCMPs and the features of TME, thereby achieving true synergy between structural design and therapeutic function.

##### Redox Response

Redox-responsive modification is generally achieved by introducing oxidation- or reduction-labile linkages into TCMPs-based carriers, thereby enabling drug release in response to the redox-imbalanced TME, which features elevated extracellular ROS levels and high intracellular glutathione (GSH) concentrations. In this strategy, TCMPs provide hydroxyl-rich matrices for chemical functionalization or crosslinking, while the introduced redox-sensitive structures serve as the key triggers for stimulus-responsive disassembly and intracellular drug release. Such modification mainly addresses the limitation that conventional NDDS often lack sufficient controllability after entering tumor tissues or cells and therefore helps improve intracellular release efficiency and antitumor efficacy.

ROS are reactive oxygen-containing molecules such as hydroxyl radicals (•OH), hydrogen peroxide (H_2_O_2_), and superoxide anions (•O_2_^−^) [98]. TME generally exhibits higher ROS levels than normal tissues as a result of abnormal metabolism. ROS-triggered systems are constructed by introducing oxidation-sensitive moieties (mainly H_2_O_2_-sensitive) such as polypropylene sulfide and borate ester bonds. The systems undergo bond cleavage or physicochemical transformation in TME, thereby achieving drug release [2]. This strategy enables selective drug release under pathological conditions while reducing premature leakage in normal tissues. Representative studies show that ROS-sensitive structures can promote the disintegration of the carriers through hydrophobic-to-hydrophilic conversion of polypropylene sulfide or induce degradation of borate ester bonds crosslinking, thereby enhancing drug release and antitumor efficacy, as demonstrated in several laminarin sulfate (LAM)-based systems and Dendrobium polysaccharides (DOP)-based systems [99,100].

However, ROS exhibits significant heterogeneity within the TME [98,101]. Different tumor cell lines vary in their capacity to produce H_2_O_2_, with some exhibiting high secretion levels and others low [102]. In addition, the concentrations of different types of ROS vary in different regions in TME, which may lead to inconsistent drug release rates. Furthermore, experiments have demonstrated that higher H_2_O_2_ concentrations correlate with greater drug release, whereas drug release is significantly low under low H_2_O_2_ conditions [99]. This heterogeneity may result in rapid carrier disintegration and drug release under high-ROS conditions, whereas carriers become trapped and fail to function under low-ROS conditions, leading to marked spatiotemporal mismatch in drug release. To address these limitations, strategies such as designing dual-responsive carriers, or applying photodynamic therapy to elevate ROS levels and induce ROS-responsive drug release, can be adopted, which hold great promise for improving the adaptability and uniform drug release of TCMPs-based carriers in heterogeneous TME.

In redox-responsive strategies, GSH-triggered systems are primarily realized by introducing reducibly cleavable chemical linkages such as disulfide bonds into TCMPs-based carriers. This strategy exploits the high-GSH intracellular microenvironment within tumor cells, enabling the responsive cleavage of these chemical bonds, thereby promoting intracellular drug release to exert antitumor efficacy. For example, disulfide bonds have been used to attach APS, BSP, and LNT to deliver antitumor components [25,34,40]. Although these systems differ in composition, all of them exploit the reductive TME to accelerate carrier degradation or swelling and improve drug release, while the intrinsic immunomodulation activities of TCMPs further cooperate with the loaded agents to enhance antitumor efficacy [40].

Diselenide bonds represent a special type of redox-responsive linkage because they can be cleaved under both oxidative and reducing conditions. Therefore, unlike conventional ROS-responsive materials that mainly rely on oxidative environments for structural disruption, diselenide-containing systems can respond to both elevated ROS and high intracellular GSH in tumor cells, thereby enabling broader redox-triggered degradation and drug release. For example, a diselenide-crosslinked ASP-based nanogel was used to co-deliver podophyllotoxin (PPT) and a cationic near-infrared (NIR) fluorescent dye IR780 [31]. In this system, the Se–Se linkage endowed the carrier with ROS/GSH dual responsiveness, evidenced by the marked degradation of its spherical nanogel structure upon exposure to 10 mM H_2_O_2_ or 10 mM DTT for 12 h.

##### Enzyme Response

Matrix metalloproteinase 2 (MMP2) is a key substance in tumor invasion and cancer progression, which can degrade nearly all proteins within the extracellular matrix (ECM). MMP2 is overexpressed in some tumor cells, especially in melanoma, thereby disrupting the histological barriers that would impede tumor cell invasion. It also modulates immune responses and promotes tumor growth [103,104]. Therefore, suppressing the overexpression of MMP2 or designing MMP2-responsive NDDS represents a therapeutic approach for treating tumors.

MMP2-responsive modification is generally achieved by introducing peptide linkers that can be specifically cleaved by MMP2 into TCMPs-based carriers. Such modification mainly addresses the limitation that TCMPs and conventional NDDS often lack sufficient selectivity toward the enzyme-rich extracellular environment of tumors, thereby improving local drug release and tumor-selective cytotoxicity. For example, an APS-based nanosystem was constructed by introducing an MMP2-responsive peptide linker, allowing enzyme overexpression in the TME to trigger selective drug release [18]. Accordingly, the cumulative release reached 74.5% within 24 h in the presence of MMP2 but remained as low as 8.7% in its absence. Meanwhile, APS not only functioned as the carrier but also contributed immunomodulatory activity and TME refinement. These properties collectively endow the nanosystem with significantly enhanced cytotoxicity in the MMP2-overexpressing TME, fully demonstrating that MMP2-responsive design can convert the disadvantage of enzyme overexpression into an advantage for responsive drug release, ultimately achieving improved antitumor efficacy.

A point worthy of attention is that, beyond MMP2, some representative enzymes within the TME, such as cathepsins and esterases, also possess considerable potential as triggers for the stimuli-responsive release of NDDS. However, current studies on enzyme-responsive systems remain only focused on MMP-2, while investigations based on other enzymatic responses are still limited. Therefore, greater efforts should be devoted to the development and exploration of diverse enzyme-responsive strategies.

Overall, the modification of TCMPs-based NDDS has substantially expanded the therapeutic potential of native polysaccharides by converting them from relatively simple biomacromolecules into multifunctional delivery platforms. Through structural and functional engineering, including hydrophobic modification, stabilization strategies, targeting design, and stimulus-responsive regulation, these systems can overcome major limitations of TCMPs and improve key aspects of tumor drug delivery, such as drug loading, colloidal stability, tumor accumulation, and controlled intracellular release. At the same time, such modifications also help preserve or amplify the intrinsic biological activities of TCMPs, allowing carrier performance and therapeutic function to be more closely integrated within a single platform. Characteristics of different modification strategies are summarized in Table 2. Notably, the design of TCMPs-based NDDS rarely relies on a single strategy. Instead, multiple approaches are often integrated within one system to achieve multifunctional performance [25,36]. This trend toward structural and functional integration is particularly of importance in the context of tumor therapy, in which treatment is increasingly shifting beyond single chemotherapy toward combination regimens involving multiple therapeutic modalities.

Although the delivery behavior of TCMPs-based NDDS is significantly improved via suitable design, the central challenge of tumor therapy is not fundamentally eliminated. Tumors are highly heterogeneous and are sustained by complex pathological networks involving drug resistance, immune evasion, and microenvironmental dysregulation. Therefore, a single therapy is often insufficient to achieve durable and comprehensive antitumor outcomes. This limitation makes combination therapy a rational and often necessary strategy for cancer treatment.

## 4. Innovative Applications of Immune-Centered Combination Therapy

Immunotherapy has emerged as an important therapeutic strategy because it can activate systemic antitumor immunity and potentially generate durable immune protection. Nevertheless, its efficacy is often restricted by insufficient antigen presentation, limited immune-cell infiltration, and strong immunosuppression within tumors. Therefore, immune-centered combination therapy provides a rational strategy: chemotherapy or phototherapy can induce tumor cell killing and antigen release, whereas immune modulation can amplify antigen presentation and T cell-mediated antitumor responses.

In this context, TCMPs-based NDDS are particularly suitable for immune-centered combination therapy because TCMPs can serve not only as drug delivery scaffolds but also as immunologically active components [37,105]. Their intrinsic immunomodulatory activities may cooperate with loaded chemotherapeutic agents, photosensitizers, or other functional molecules, while rational platform engineering can further improve tumor accumulation, responsive release, and multimodal coordination. Thus, the value of TCMPs-based NDDS lies not merely in passive drug delivery but in their ability to integrate carrier performance, immune regulation, and therapeutic synergy within a single platform. Although related studies have gradually increased, the mechanistic integration between precise structural modification and immune-centered combination therapy remains insufficient. Accordingly, this chapter focuses on representative TCMPs-based NDDS used in chemo-immunotherapy, photo-immunotherapy, and chemo-photo-immunotherapy (Table 3).

### 4.1. Traditional Chinese Medicine Polysaccharides-Based Drug Delivery Systems for Chemo-Immunotherapy

Given that many TCMPs possess inherent immunomodulatory properties, NDDS based on TCMPs offer unique advantages for the synergistic integration of chemotherapy and immunotherapy. In such systems, chemotherapeutic agents mediate direct tumor cell killing and may induce immunogenic cell death (ICD), whereas TCMPs can further amplify antitumor immunity by promoting antigen presentation, alleviating immunosuppression, and enhancing immune cell infiltration. As a result, TCMPs-based platforms have attracted increasing interest as multifunctional systems capable of combining drug delivery with immune intervention.

Current TCMPs-based chemo-immunotherapeutic systems generally share several common design features. First, TCMPs such as ASP, APS, and GLP can serve as carrier materials for chemotherapeutic agents while retaining intrinsic immunomodulatory activity, thus enabling the functional integration of cytotoxic drug delivery and immune regulation. Second, some systems exploit tumor-associated features, such as MMP2 overexpression, to achieve tumor-responsive drug release, thereby improving local chemotherapy and reducing premature leakage and off-target exposure. Third, most systems are designed to couple chemotherapy-induced tumor cell killing with immune activation.

These general design principles are well reflected in several representative TCMPs-based chemo-immunotherapeutic systems. An APS-based nanosystem loaded with PTX represents a typical example of this integrated strategy [24]. This system actively targeted 4T1 tumor cells through GLUT1-mediated uptake, while PTX induced immunogenic cell death and APS further promoted BMDC maturation. APS also downregulated PD-L1 expression through the AKT/mTOR pathway, thereby attenuating immune escape. As a result, the nanosystem increased intratumoral CD8^+^ T-cell infiltration, achieved a tumor inhibition rate of 92.28%, and completely suppressed pulmonary metastasis in the model. This study shows that APS combines carrier functions and immunoregulatory activity, thus further extending chemotherapy-induced immune activation into a broader antitumor response.

Rational modification of TCMPs can enhance delivery performance without abolishing their intrinsic immunological activity, thereby making TCMPs-based systems more effective and multifunctional in chemo-immunotherapy. Taking the MMP2-responsive delivery system AP-PP-DOX as an example, this system enables the specific release of DOX in the TME while fully retaining the intrinsic biological activity of the APS fragments, ultimately achieving synergistic enhancement of chemotherapy and immunomodulation by restoring the immune balance between T helper 1 cells (Th1) and T helper 2 cells (Th2) and promoting intratumoral T-cell infiltration [18]. Likewise, the self-assembly ability of GLP was enhanced after being sulfated, and gefitinib and DOX were co-delivered [38]. The system showed strengthened immunoregulatory effects, as evidenced by increased dendritic cells (DCs) activation, enhanced macrophage phagocytosis, promoted M1 polarization, facilitated CD47 internalization, and an elevated M1/M2 ratio in the TME. These findings suggest that structural modification not only improves the efficiency of TCMPs-based delivery systems but also preserves or amplifies polysaccharide-mediated immune regulation, thereby conferring additional benefits for combination therapy.

Overall, the value of TCMPs-based systems in chemo-immunotherapy lies in their ability to integrate efficient chemotherapeutic drug delivery, tumor-responsive release, and immune modulation within a single platform. Through such design, chemotherapy serves not only as a direct tumor-killing modality, but also as a trigger for subsequent immune activation, whereas TCMPs further amplify and sustain the resulting antitumor response.

### 4.2. Traditional Chinese Medicine Polysaccharides-Based Drug Delivery Systems for Photo-Immunotherapy

As a non-invasive therapy, phototherapy has been widely used for tumor treatment in recent years, with photothermal therapy (PTT) and photodynamic therapy (PDT) being the most common approaches [108]. PTT employs photothermal converters to transform light energy into heat energy, causing irreversible thermal damage to tumor cells, while PDT selectively damages tumor cells and tumor microvasculature by generating reactive oxygen species [109]. Although phototherapy has clear advantages in spatial–temporal control and local tumor ablation, its antitumor efficacy often remains incomplete when used alone because photoinduced tumor destruction does not automatically translate into durable immune protection [110]. Although PDT or PTT may trigger ICD and lead to the release of tumor antigens and damage-associated molecular patterns (DAMPs), these immune-relevant signals are often rapidly cleared or insufficiently presented to antigen-presenting cells. Accordingly, the core task of TCMPs-based phototherapy-immunomodulatory platforms is to build a material environment that captures, preserves, and biologically amplifies the immune consequences of photoinduced tumor injury.

**Figure 4:**
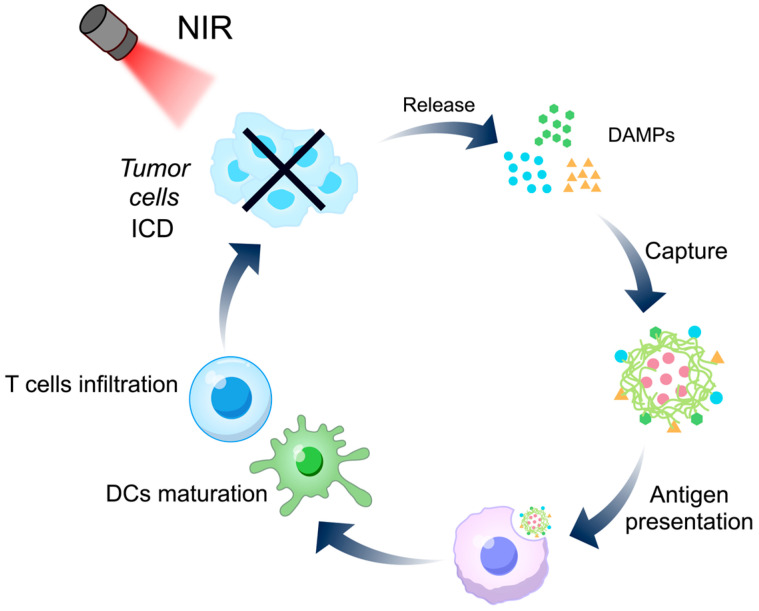
Mechanism of TCMPs-based NDDS in photo-immunotherapy. Phototherapy induces ICD and DAMPs release from tumor cells. TCMPs-based NDDS capture these signals and are phagocytosed by macrophages to activate cellular immunity, also promoting DC maturation and T cell infiltration.

In detail, NIR first induces ICD of tumor cells, causing the release of DAMPs or tumor autoantigens. Subsequently, TCMPs-based NDDS further exert the effects of capturing, retaining and amplifying the presentation of these immune signals, thereby promoting the maturation of DCs and ultimately converting the local effects induced by phototherapy into a stronger T cell-mediated systemic anti-tumor immunity (Figure 4). In this process, relying on their inherent immunomodulatory activity, TCMPs reconstruct the temporary and local damage triggered by phototherapy into a cascade immune response. For example, Tao et al. constructed a DOP-based photodynamic system in which the DOP were modified with cholesterol to formulate amphiphilic DOP and improve their self-assembly efficiency [106]. The positively charged nanoparticles captured negatively charged DAMPs released after PDT, thereby reducing their rapid degradation and improving the efficiency of their uptake by DCs and subsequent transport to draining lymph nodes. Meanwhile, DOP itself possesses the ability to promote DC maturation. Thus, the unique feature of this system lies in the integration of antigen capture and polysaccharide-mediated endogenous immune stimulation into a single nanocarrier.

More broadly, phototherapy combined with immune activation may represent one of the most conceptually compatible application scenarios for TCMPs-based NDDS. This is because the central bottleneck of phototherapy is often not insufficient local damage but insufficient translation of that damage into systemic immune benefit. TCMPs are well positioned to address this bottleneck because they can simultaneously provide structural adaptability, high biocompatibility, and intrinsic immunological activity.

Beyond the dual-modal systems discussed above, TCMPs-based NDDS have also begun to show potential for integrating chemotherapy, phototherapy, and immune activation within a single platform, indicating a further evolution of combination therapy toward higher-order multimodal coordination [31,107]. In such systems, TCMPs serve also as the structural and functional basis for coupling cytotoxic treatment with immune regulation. This integrated design may facilitate a more effective transition from local tumor destruction to systemic antitumor immune activation. Although current studies remain scarce and immunological validation is still insufficient, these preliminary findings nonetheless highlight an emerging trend toward more sophisticated and immunologically coordinated TCMPs-based therapeutic platforms.

## 5. Conclusions

Traditional Chinese medicine polysaccharides have shown broad potential as the carriers of anticancer drugs because of their good biocompatibility, structural diversity, and intrinsic pharmacological activities. As summarized in this review, TCMPs-based NDDS can not only improve drug loading, stability, and tumor accumulation through appropriate modification strategies but also contribute to antitumor therapy through immunomodulation and synergistic effects with loaded agents. These characteristics enable TCMPs-based systems to address some limitations of conventional nanocarriers. Current studies have demonstrated that property-enhancing modification and functional-oriented modification are effective approaches to enhance the delivery performance of TCMPs-based NDDS. Moreover, the application of TCMPs-based platforms in combination therapies, such as chemotherapy combined with immunotherapy, has further improved therapeutic efficacy in preclinical models.

Despite these encouraging results, several key bottlenecks for clinical translation persist. The natural heterogeneity and structural complexity of polysaccharides complicate precise structure–function correlation, which may affect reproducibility and rational system design. In addition, the structure–function basis underlying the carrier performance of TCMPs remains insufficiently understood, and this will affect the applications of TCMPs as carriers. Moreover, the application of precisely modified TCMPs-based NDDS in combination therapy, especially in multi-model combination therapy, is insufficient, which may restrict the full use of the NDDS. These issues represent important considerations for the further development of TCMPs-based NDDS.

From a future perspective, future development should focus on four interrelated aspects. First, it is necessary to establish standardized upstream processes in order to reduce batch heterogeneity and improve the reproducibility and comparability of studies. Second, further investigations into the structure–activity relationship of TCMPs as carriers are required to guide the subsequent modification and design principles of TCMPs-based NDDS. Third, the immunity enhancing property of TCMPs lays a robust foundation for their applications in immunotherapy; thus, further exploration of these materials in immunotherapy is warranted, with particular emphasis on polysaccharide components suitable for constructing delivery carriers like fungal medicine polysaccharides [111]. Finally, there is an urgent need to explore the deeper potential of precisely modified TCMPs-based NDDS in combination therapy, as well as how different TCMPs shape the immune microenvironment and determine the final antitumor effects of NDDS.

## Figures and Tables

**Table 1:** Main structural features, molecular weight range, intrinsic bioactivities, and applications in tumor drug delivery of representative TCMPs.

Polysaccharides	Main Structural Features	Molecular Weight Range (kDa)	Intrinsic Bioactivities	Applications in Tumor Drug Delivery
*Astragalus* Polysaccharides (APS)	Glucose, galactose, etc.; β-(1,4), α-(1,6), etc.; hydroxyl, etc.	~10–500 [22]	Strong immunomodulatory activity, anti-inflammatory, and metabolic regulation; can act as both carrier and immune regulator; potential active uptake in 4T1/TNBC cells	Used as immunoactive carriers or carrier components for PTX, DOX, Cur, and multifunctional chemo-immunotherapeutic platforms, including APS-PTX NPs, AP-PP-DOX, GACS-Cur@RBCm, and QDFA@Cur [18,23,24,25].
*Angelica sinensis* Polysaccharides (ASP)	Glucose, rhamnose, etc.; β-(1,4), β-(1,6), etc.; hydroxyl, carboxyl, etc.	~20–700 [26]	Hematopoietic regulation, antioxidant, immunomodulatory, and antitumor activity; intrinsic liver-targeting capability through ASGPR recognition	Used in liver-targeted and multifunctional systems, including ASP-DOCA, ASP-BBR-PM@HNK, BAI@ASPOBA, and IR780@PPTASP [27,28,29,30,31].
*Bletilla striata* Polysaccharides (BSP)	Glucose, mannose, etc.; β-(1,4), β-(1,6), etc.; hydroxyl, acetyl, etc.	~150–500 [32]	Immunomodulatory, antioxidant, anti-inflammatory, and antitumor activity; excellent film-forming/adhesion; good biocompatibility, low immunogenicity	Used for hydrophobic-drug delivery and targeted/redox-responsive nanocarriers, including SA-BSP, FA-BSP-SA, and BSP-SS-SA systems [15,16,33,34].
*Ganoderma lucidum* Polysaccharides (GLP)	Glucose, etc.; β-(1,3), β-(1,6), etc.; hydroxyl, acetyl, etc.	~1–700 [35]	Immune activation and antitumor support	Used as immunoactive components or modified carriers in RCGDDH NPs, GLP-Au, sulfated GLP-based GEF/DOX co-delivery systems, and GLP-modified photothermal platforms [36,37,38].
Lentinan (LNT)	Glucose, etc.; β-(1,3), α-(1,6), etc.; only hydroxyl	~300–2000 [39]	Potent immunomodulatory and antitumor activity; classic immune adjuvant; structure supports bioactivity	Used as immunoactive carrier or conjugated scaffold in LNT-DOX, LDD NGs, MWNTs-Ge-Le systems, and LNT-UA [17,40,41,42].
*Lycium barbarum* polysaccharides (LBP)	Arabinose, galactose, galacturonic acid, etc.; β-(1→3), β-(1→6), etc.; hydroxyl, uronic carboxyl, etc.	~20–1000 [43]	Immunomodulatory, antioxidant, anti-inflammatory, and antitumor activities; form ionically crosslinked networks	Used in polysaccharide-based hydrogels and responsive delivery matrices, including DOX-conjugated or pH-responsive LBP-based delivery platforms [44,45].
Ginseng polysaccharides (GP)	Glucose, galactose, etc.; α-(1→4), β-(1,3), etc.; hydroxyl and uronic carboxyl, etc.	~1–400 [46]	Antitumor, immunomodulatory, antioxidant, and hypoglycemic activities	\
*Poria cocos* polysaccharides (PCP)	Glucose, arabinose, etc.; β-(1→3), β-(1→6), etc.; hydroxyl, etc.	~40–5000 [47]	Antitumor, immunomodulatory, and antioxidant activities	\

**Table 2:** Modification strategies of TCMPs-based drug delivery systems and their structure–function rationale in tumor therapy.

Modification Strategy	Structural Basis of TCMPs	Introduced Moiety or Linkage	Main Design Purpose	Improved Delivery Property
Hydrophobic modification	Abundant hydroxyl groups and strong intrinsic hydrophilicity	Hydrophobic moieties such as SA and DOCA, usually introduced through esterification	Convert hydrophilic polysaccharides into amphiphilic derivatives	Improved self-assembly, micelle formation, hydrophobic drug encapsulation, and sustained release
Bio-membrane coating	\	RBCm coatings	Shield carriers from immune recognition and prolong circulation	Improved colloidal stability, reduced clearance, enhanced in vivo persistence, and better tissue accumulation
Active targeting modification	Abundant hydroxyl groups; partial intrinsic recognition properties of some TCMPs	Folate, cationic mitochondria-targeting groups, or other targeting ligands	Improve tumor-cell or subcellular recognition	Enhanced tumor accumulation, cellular uptake, and organelle-specific delivery
pH-responsive design	Abundant hydroxyl groups	Ester bonds, Schiff base bonds, and borate ester bonds	Exploit acidic TME	Acid-triggered carrier dissociation or drug release
Redox-responsive design	Abundant hydroxyl groups	Disulfide bonds, diselenide bonds, borate ester-containing structures, or ROS-sensitive motifs	Respond to high intracellular GSH or elevated ROS levels in tumor tissues	Stimulus-triggered degradation, swelling, or intracellular drug release
Enzyme-responsive design	Abundant hydroxyl groups	MMP2-cleavable peptide linkers or other enzyme-sensitive motifs	Use tumor-associated enzyme overexpression to trigger local release	Enzyme-triggered drug release and improved tumor selectivity

**Table 3:** Representative TCMPs-based platforms for immunotherapy-centered combination therapy in tumor treatment.

Modality	TCMPs-Based Platforms	Cargo(s)/Therapeutic Component(s)	Key Design Feature	In Vivo Tumor Models	Main Therapeutic Outcome	Ref.
Chemo-immunotherapy	APS-PTX	PTX	Native APS-based nanoplatform with GLUT1-mediated uptake by 4T1 cells	In situ 4T1 hormonal mouse model	Achieved a tumor inhibition rate of 92.28% and complete suppression of pulmonary metastasis	[24]
	AP-PP-DOX	DOX	APS-based system incorporating an MMP2-responsive peptide linker for tumor-selective release	\	Restored the Th1/Th2 balance and promoted intratumoral T-cell infiltration	[18]
	Sulfated GLP-based co-delivery system	Gefitinib + DOX	Sulfation-enhanced GLP self-assembly enabling dual-drug co-delivery	Murine CT26 colorectal cancer peritoneal metastasis model	Enhanced DCs activation, macrophage phagocytosis, and M1 polarization	[38]
Photo-immunotherapy	DOP@BCP	A positively charged photosensitizer TPA-3BCP	Cholesterol grafting generated amphiphilic DOP nanoparticles with improved self-assembly and DAMPs-capturing capability	Bilateral subcutaneous CT26 tumor model	Strengthened PDT-triggered immune activation by promoting DAMPs capture, DCs uptake, lymph-node transport, and DCs maturation	[106]
Chemo-photo-immunotherapy	IR780@PPTASP	PPT + IR780	ASP-based nanogel crosslinked by diselenide bonds with ROS/GSH dual responsiveness	Zebrafish xenograft model	Enabled integrated chemotherapy, phototherapy, and immune activation within a single redox-responsive platform	[31]
	GLP-LU-TeNRs	Luteolin + tellurium nanorods	GLP and luteolin were used as modifiers and stabilizers to controllably fabricate tellurium nanorods with uniform morphology	Zebrafish xenograft model	Exhibited excellent photothermal properties and colloidal stability and showed significant antitumor effects both in vitro and in vivo	[107]

## Data Availability

This study did not involve the generation or analysis of any datasets.

## Abbreviations

The following abbreviations are used in this manuscript:

APS	*Astragalus* polysaccharides
ASGPR	Asialoglycoprotein receptor
ASP	*Angelica sinensis* polysaccharides
BAI	Baicalein
BBR	Berberine
BSP	*Bletilla striata* polysaccharides
Cur	Curcumin
DAMPs	Damage-associated molecular patterns
DB	Degree of branching
DCs	Dendritic cells
DOCA	Deoxycholic acid
DOP	*Dendrobium* polysaccharides
DOX	Doxorubicin
DS	Degree of substitution
DTX	Docetaxel
ECM	Extracellular matrix
FA	Folate
FRs	Folate receptors
GLP	*Ganoderma lucidum* polysaccharides
GMA	Glycidyl methacrylate
GP	Ginseng polysaccharides
GSH	Glutathione
H_2_O_2_	Hydrogen peroxide
ICD	Immunogenic cell death
LAM	Laminarin sulfate
LBP	*Lycium barbarum* polysaccharides
LNT	Lentinan
MMP2	Matrix metalloproteinase 2
NDDS	Nano-drug delivery systems
NIR	Near-infrared
PCP	*Poria cocos* polysaccharides
PDI	Polydispersity index
PDT	Photodynamic therapy
PPT	Podophyllotoxin
PTT	Photothermal therapy
RBC	Red blood cell
RBCm	Red blood cell membrane
ROS	Reactive oxygen species
SA	Stearic acid
Th1	T helper 1 cells
Th2	T helper 2 cells
TCM	Traditional Chinese medicine
TCMPs	Traditional Chinese medicine polysaccharides
TME	Tumor microenvironment
•OH	Hydroxyl radicals
•O_2_^−^	Superoxide anions

## References

[1] Gilbertson R.J. (2011). Mapping cancer origins. Cell.

[2] Meng Q., Hu H., Jing X., Sun Y., Zhou L., Zhu Y., Yu B., Cong H., Shen Y. (2021). A modular ROS-responsive platform co-delivered by 10-hydroxycamptothecin and dexamethasone for cancer treatment. J. Control. Release.

[3] Patra J.K., Das G., Fraceto L.F., Campos E.V.R., Rodriguez-Torres M.d.P., Acosta-Torres L.S., Diaz-Torres L.A., Grillo R., Swamy M.K., Sharma S. (2018). Nano based drug delivery systems: Recent developments and future prospects. J. Nanobiotechnol..

[4] Xu Z., Xie Y., Chen W., Deng W. (2025). Nanocarrier-based systems for targeted delivery: Current challenges and future directions. MedComm.

[5] Guo S., Huang L. (2014). Nanoparticles containing insoluble drug for cancer therapy. Biotechnol. Adv..

[6] Gomerdinger V.F., Nabar N., Hammond P.T. (2025). Advancing engineering design strategies for targeted cancer nanomedicine. Nat. Rev. Cancer.

[7] Wang M., Deng W., Fu M., Cao X., Yang Y., Su W., Yu J., Xu X. (2011). Efficient gene transfer into rat mesenchymal stem cells with cationized *Lycium barbarum* polysaccharides nanoparticles. Carbohydr. Polym..

[8] Tzianabos A.O. (2000). Polysaccharide immunomodulators as therapeutic agents: Structural aspects and biologic function. Clin. Microbiol. Rev..

[9] Xiao Z., Guo Y., Li J., Jiang X., Wu F., Wang Y., Zhang Y., Zhou W. (2024). Harnessing traditional Chinese medicine polysaccharides for combatting COVID-19. Carbohydr. Polym..

[10] Xie L., Shen M., Hong Y., Ye H., Huang L., Xie J. (2020). Chemical modifications of polysaccharides and their anti-tumor activities. Carbohydr. Polym..

[11] Pan Y., Wang C., Chen Z., Li W., Yuan G., Chen H. (2017). Physicochemical properties and antidiabetic effects of a polysaccharide from corn silk in high-fat diet and streptozotocin-induced diabetic mice. Carbohydr. Polym..

[12] Wang B., Wang X., Xiong Z., Lu G., Ma W., Lv Q., Wang L., Jia X., Feng L. (2022). A review on the applications of Traditional Chinese medicine polysaccharides in drug delivery systems. Chin. Med..

[13] Yang Y., Yu M., Mo Y., Cheng Y., Huang B., Wang W., Zhu M., Jia X., Feng L., Yang B. (2024). Metal-ion-binding properties of glycyrrhiza polysaccharide extracted from *licorice*: Structural characterization and potential application in drug delivery. Carbohydr. Polym..

[14] Xiong W., Li L., Wang Y., Yu Y., Wang S., Gao Y., Liang Y., Zhang G., Pan W., Yang X. (2016). Design and evaluation of a novel potential carrier for a hydrophilic antitumor drug: *Auricularia auricular* polysaccharide-chitosan nanoparticles as a delivery system for doxorubicin hydrochloride. Int. J. Pharm..

[15] Guan Q., Zhang G., Sun D., Wang Y., Liu K., Wang M., Sun C., Zhang Z., Li B., Lv J. (2017). In vitro and in vivo evaluation of docetaxel-loaded stearic acid-modified *Bletilla striata* polysaccharide copolymer micelles. PLoS ONE.

[16] Zhang G., Huang L., Wu J., Liu Y., Zhang Z., Guan Q. (2020). Doxorubicin-loaded folate-mediated pH-responsive micelle based on *Bletilla striata* polysaccharide: Release mechanism, cellular uptake mechanism, distribution, pharmacokinetics, and antitumor effects. Int. J. Biol. Macromol..

[17] Wang Y., Chen J., Han Q., Luo Q., Zhang H., Wang Y. (2019). Construction of doxorubicin-conjugated lentinan nanoparticles for enhancing the cytotoxocity effects against breast cancer cells. Colloids Surf. A Physicochem. Eng. Asp..

[18] Wang M.-Z., He X., Yu Z., Wu H., Yang T.-H. (2020). A nano drug delivery system based on *Angelica sinensis* polysaccharide for combination of chemotherapy and immunotherapy. Molecules.

[19] Huang Y., Shi F., Wang L., Yang Y., Khan B.M., Cheong K.-L., Liu Y. (2019). Preparation and evaluation of *Bletilla striata* polysaccharide/carboxymethyl chitosan/Carbomer 940 hydrogel for wound healing. Int. J. Biol. Macromol..

[20] Yang J., Lin J., Zhang J., Chen X., Wang Y., Shen M., Xie J. (2022). Fabrication of zein/*mesona chinensis* polysaccharide nanoparticles: Physical characteristics and delivery of quercetin. ACS Appl. Bio Mater..

[21] Sun L., Liu Y., Sun Q., Wang G., Du B., Liu B., Gao T., Zhao P., Yang Y., Rong R. (2025). Polysaccharides from traditional Chinese medicine and their nano-formulated delivery systems for cancer immunotherapy. Carbohydr. Polym..

[22] Jin M., Zhao K., Huang Q., Shang P. (2014). Structural features and biological activities of the polysaccharides from *Astragalus membranaceus*. Int. J. Biol. Macromol..

[23] Guo C., Hou X., Liu Y., Zhang Y., Xu H., Zhao F., Chen D. (2021). Novel Chinese *angelica* polysaccharide biomimetic nanomedicine to curcumin delivery for hepatocellular carcinoma treatment and immunomodulatory effect. Phytomedicine.

[24] Lan J., Nie W., Bi Z., Zeng R., Li Z., Zhang T., Ding Y. (2025). *Astragalus* polysaccharide-based nano-platforms loading PTX to boost chemo-immunotherapy for triple-negative breast cancer with intrinsic GLUT-targeting ability and immunoregulatory activity. J. Nanobiotechnol..

[25] Wang B., Guo C., Liu Y., Han G., Li Y., Zhang Y., Xu H., Chen D. (2020). Novel nano-pomegranates based on *astragalus* polysaccharides for targeting ERα-positive breast cancer and multidrug resistance. Drug Deliv..

[26] Chen Z., Cheng L., He Y., Wei X. (2018). Extraction, characterization, utilization as wound dressing and drug delivery of *Bletilla striata* polysaccharide: A review. Int. J. Biol. Macromol..

[27] Zhang Y., Cui Z., Mei H., Xu J., Zhou T., Cheng F., Wang K. (2019). *Angelica sinensis* polysaccharide nanoparticles as a targeted drug delivery system for enhanced therapy of liver cancer. Carbohydr. Polym..

[28] Sun H., Nai J., Deng B., Zheng Z., Chen X., Zhang C., Sheng H., Zhu L. (2024). *Angelica sinensis* polysaccharide-based nanoparticles for liver-targeted delivery of oridonin. Molecules.

[29] Wang B., Lv B., Li H., Zhang J., Ding Y., Zhou J., Bu M., Fan L., Han C. (2025). Design of self-assembled micelles based on natural dual-targeting strategies and evaluation of their anti-liver cancer effects as drug delivery systems. npj Precis. Oncol..

[30] Wang S., Nie F., Lin Z., Xu J., Guo Y. (2025). Natural polysaccharide-small molecule smart responsive nanogels: Design, synthesis, and synergistic chemoimmunotherapy for tumors. Int. J. Biol. Macromol..

[31] Wang S., Cao R., Lin Z., He W., Liao H., Xu J., Guo Y. (2025). Biocompatible *Astragalus* polysaccharide-based nanogels for oncology: Synthesis, characterization, and therapeutic potential. Int. J. Biol. Macromol..

[32] Chen H., Zeng J., Wang B., Cheng Z., Xu J., Gao W., Chen K. (2021). Structural characterization and antioxidant activities of *Bletilla striata* polysaccharide extracted by different methods. Carbohydr. Polym..

[33] Zhao L., Sun D., Lu H., Han B., Zhang G., Guan Q. (2018). In vitro characterization of pH-sensitive *Bletilla Striata* polysaccharide copolymer micelles and enhanced tumour suppression in vivo. J. Pharm. Pharmacol..

[34] Liu Y., Sun C., Zhang G., Wu J., Huang L., Qiao J., Guan Q. (2020). Bio-responsive *Bletilla striata* polysaccharide-based micelles for enhancing intracellular docetaxel delivery. Int. J. Biol. Macromol..

[35] Ferreira I.C., Heleno S.A., Reis F.S., Stojkovic D., Queiroz M.J.R., Vasconcelos M.H., Sokovic M. (2015). Chemical features of *Ganoderma* polysaccharides with antioxidant, antitumor and antimicrobial activities. Phytochemistry.

[36] Zheng D., Zhao J., Tao Y., Liu J., Wang L., He J., Lei J., Liu K. (2020). pH and glutathione dual responsive nanoparticles based on *Ganoderma lucidum* polysaccharide for potential programmable release of three drugs. Chem. Eng. J..

[37] Zhang S., Pang G., Chen C., Qin J., Yu H., Liu Y., Zhang X., Song Z., Zhao J., Wang F. (2019). Effective cancer immunotherapy by *Ganoderma lucidum* polysaccharide-gold nanocomposites through dendritic cell activation and memory T cell response. Carbohydr. Polym..

[38] Pang G., Wei S., Zhao J., Wang F.-J. (2023). Improving nanochemoimmunotherapy efficacy by boosting “eat-me” signaling and downregulating “don’t-eat-me” signaling with *Ganoderma lucidum* polysaccharide-based drug delivery. J. Mater. Chem. B.

[39] Zhang Y., Li S., Wang X., Zhang L., Cheung P.C. (2011). Advances in lentinan: Isolation, structure, chain conformation and bioactivities. Food Hydrocoll..

[40] Wang S., Nie F., Lin Z., Cao R., Xu J., Guo Y. (2024). Construction of an innovative nanogel and its applications for achieving chemo-immunotherapy of tumors. ACS Appl. Mater. Interfaces.

[41] Zhang P., Yi W., Hou J., Yoo S., Jin W., Yang Q. (2018). A carbon nanotube-gemcitabine-lentinan three-component composite for chemo-photothermal synergistic therapy of cancer. Int. J. Nanomed..

[42] Mao Q., Min J., Zeng R., Liu H., Li H., Zhang C., Zheng A., Lin J., Liu X., Wu M. (2022). Self-assembled traditional Chinese nanomedicine modulating tumor immunosuppressive microenvironment for colorectal cancer immunotherapy. Theranostics.

[43] Masci A., Carradori S., Casadei M.A., Paolicelli P., Petralito S., Ragno R., Cesa S. (2018). *Lycium barbarum* polysaccharides: Extraction, purification, structural characterisation and evidence about hypoglycaemic and hypolipidaemic effects. A review. Food Chem..

[44] Zhang J., Bao C.J., Zhang X.L., Ren Y.H., Zhao W., Zhang F., Guo S., Duan J.A., Duan J.L., Han X. (2026). *Lycium Barbarum* Polysaccharide-based Hydrogel as a Novel Oral Colon-Targeted Drug Delivery System for Enhanced MSS-CRC Treatment by Macrophage Lipid Reprogramming. Adv. Funct. Mater..

[45] Wang Y., Bai F., Luo Q., Wu M., Song G., Zhang H., Cao J., Wang Y. (2019). *Lycium barbarum* polysaccharides grafted with doxorubicin: An efficient pH-responsive anticancer drug delivery system. Int. J. Biol. Macromol..

[46] Guo M., Shao S., Wang D., Zhao D., Wang M. (2021). Recent progress in polysaccharides from *Panax ginseng CA Meyer*. Food Funct..

[47] Li X., He Y., Zeng P., Liu Y., Zhang M., Hao C., Wang H., Lv Z., Zhang L. (2019). Molecular basis for *Poria cocos* mushroom polysaccharide used as an antitumour drug in China. J. Cell. Mol. Med..

[48] Hou H., Li L., Islam T., Shi J., Hou M., Jiang S., Zhao X., Lin C., Han Q., Zhu L. (2026). Precise structural characterization and therapeutic potential of a glucomannan polysaccharide from dried rhizome of plant *Bletilla striata* against ulcerative colitis. Carbohydr. Polym..

[49] Li X., Rao Z., Xie Z., Qi H., Zeng N. (2022). Isolation, structure and bioactivity of polysaccharides from *Atractylodes macrocephala*: A review. J. Ethnopharmacol..

[50] Ma W., Zhou Y., Lou W., Wang B., Li B., Liu X., Yang J., Yang B., Liu J., Di D. (2022). Mechanism regulating the inhibition of lung cancer A549 cell proliferation and structural analysis of the polysaccharide *Lycium barbarum*. Food Biosci..

[51] Wu D.-T., Zhao Y.-X., Guo H., Gan R.-Y., Peng L.-X., Zhao G., Zou L. (2021). Physicochemical and biological properties of polysaccharides from *Dictyophora indusiata* prepared by different extraction techniques. Polymers.

[52] Zhang C., Tang L., Su X., Li Q., Guo H., Liu Z., Wei Z., Wang F. (2023). Research on the impact of deep eutectic solvent and hot-water extraction methods on the structure of *Polygonatum sibiricum* polysaccharides. Molecules.

[53] Lupo C., Boulos S., Nyström L. (2020). Influence of partial acid hydrolysis on size, dispersity, monosaccharide composition, and conformation of linearly-branched water-soluble polysaccharides. Molecules.

[54] Jing Y., Yan M., Zhang H., Liu D., Qiu X., Hu B., Zhang D., Zheng Y., Wu L. (2023). Effects of extraction methods on the physicochemical properties and biological activities of polysaccharides from *Polygonatum sibiricum*. Foods.

[55] Zhang Y., Zhou T., Wang H., Cui Z., Cheng F., Wang K.-P. (2016). Structural characterization and in vitro antitumor activity of an acidic polysaccharide from *Angelica sinensis (Oliv.) Diels*. Carbohydr. Polym..

[56] Nai J., Zhang C., Shao H., Li B., Li H., Gao L., Dai M., Zhu L., Sheng H. (2021). Extraction, structure, pharmacological activities and drug carrier applications of *Angelica sinensis* polysaccharide. Int. J. Biol. Macromol..

[57] Guo M.Q., Hu X., Wang C., Ai L. (2017). Polysaccharides: Structure and solubility. Solubility of Polysaccharides.

[58] Wang Y., Liu Y., Yu H., Zhou S., Zhang Z., Wu D., Yan M., Tang Q., Zhang J. (2017). Structural characterization and immuno-enhancing activity of a highly branched water-soluble β-glucan from the spores of *Ganoderma lucidum*. Carbohydr. Polym..

[59] Banerjee R., Kumar K.J., Kennedy J.F. (2023). Structure and drug delivery relationship of acidic polysaccharides: A review. Int. J. Biol. Macromol..

[60] Huang F., Fan Y., Liu X., Chen Y., Huang Y., Meng Y., Liang Y. (2024). Structural characterization and innate immunomodulatory effect of glucomannan from *Bletilla striata*. Int. J. Biol. Macromol..

[61] Yue L., Wang W., Wang Y., Du T., Shen W., Tang H., Wang Y., Yin H. (2016). *Bletilla striata* polysaccharide inhibits angiotensin II-induced ROS and inflammation via NOX4 and TLR2 pathways. Int. J. Biol. Macromol..

[62] Wang J., Ge B., Li Z., Guan F., Li F. (2016). Structural analysis and immunoregulation activity comparison of five polysaccharides from *Angelica sinensis*. Carbohydr. Polym..

[63] Yu F., Li H., Meng Y., Yang D. (2013). Extraction optimization of *Angelica sinensis* polysaccharides and its antioxidant activity in vivo. Carbohydr. Polym..

[64] Zhang Y., Ji W., Qin H., Chen Z., Zhou Y., Zhou Z., Wang J., Wang K. (2025). *Astragalus* polysaccharides alleviate DSS-induced ulcerative colitis in mice by restoring SCFA production and regulating Th17/Treg cell homeostasis in a microbiota-dependent manner. Carbohydr. Polym..

[65] Ye M., Fan M., Zhao Y., Wang F., Yang X., Yao W., Gao X., Yu J., Liu W. (2025). Low molecular weight *Astragalus membranaceus* polysaccharides alleviates dextran sulfate sodium-induced colitis in mice. Carbohydr. Polym..

[66] Liu C., Dai K.-Y., Ji H.-Y., Jia X.-Y., Liu A.-J. (2022). Structural characterization of a low molecular weight *Bletilla striata* polysaccharide and antitumor activity on H22 tumor-bearing mice. Int. J. Biol. Macromol..

[67] Liu W., Li W., Sui Y., Li X.-Q., Liu C., Jing H., Zhang H., Cao W. (2019). Structure characterization and anti-leukemia activity of a novel polysaccharide from *Angelica sinensis (Oliv.) Diels*. Int. J. Biol. Macromol..

[68] Wang W., He S., Hong T., Zhang Y., Sui H., Zhang X., Ma Y. (2017). Synthesis, self-assembly, and in vitro toxicity of fatty acids-modified *Bletilla striata* polysaccharide. Artif. Cells Nanomed. Biotechnol..

[69] Liu M., Zhang Z.-X., Wang J.-H., Guo R.-B., Zhang L., Kong L., Yu Y., Zang J., Liu Y., Li X.-T. (2025). Immunomodulatory and anti-ovarian cancer effects of novel *astragalus* polysaccharide micelles loaded with podophyllotoxin. Int. J. Biol. Macromol..

[70] Li W., Gong H., Yan S., Fu Y., Huang J., Mei Y., Wang Y. (2025). Effective Delivery of Rutin Through *Astragalus* Polysaccharides Micelles for Downregulating PD-L1 by Inhibiting Matrix Metalloproteinases. ACS Appl. Mater. Interfaces.

[71] Hou Y., Wu J., Jin B., Zhang Y., Zhang J., Zhang Y., Gao J., Sun X., Dong Z. (2025). Preparation of hierarchically targetable *Astragalus* polysaccharide lipid nanoparticles and study on their therapeutic effect on orthotopic colon cancer. Mater. Today Bio.

[72] Zhao T., Dong S., Shao S., Lu G., Hou J., Yang F. (2023). Injectable hydroethanolic physical gels based on *Codonopsis pilosula* polysaccharide for sustained anticancer drug delivery. Int. J. Biol. Macromol..

[73] Jang H., Zhi K., Wang J., Zhao H., Li B., Yang X. (2020). Enhanced therapeutic effect of paclitaxel with a natural polysaccharide carrier for local injection in breast cancer. Int. J. Biol. Macromol..

[74] D’Souza A.A., Devarajan P.V. (2015). Asialoglycoprotein receptor mediated hepatocyte targeting—Strategies and applications. J. Control. Release.

[75] Liu X., Wu Z., Guo C., Guo H., Su Y., Chen Q., Sun C., Liu Q., Chen D., Mu H. (2022). Hypoxia responsive nano-drug delivery system based on *angelica* polysaccharide for liver cancer therapy. Drug Deliv..

[76] Surenjav U., Zhang L., Xu X., Zhang X., Zeng F. (2006). Effects of molecular structure on antitumor activities of (1→3)-β-D-glucans from different *Lentinus edodes*. Carbohydr. Polym..

[77] Tu L., Xing B., Ma S., Zou Z., Wang S., Feng J., Cheng M., Jin Y. (2025). A review on polysaccharide-based tumor targeted drug nanodelivery systems. Int. J. Biol. Macromol..

[78] Han B., Baruah K., Cox E., Vanrompay D., Bossier P. (2020). Structure-functional activity relationship of β-glucans from the perspective of immunomodulation: A mini-review. Front. Immunol..

[79] Demleitner S., Kraus J., Franz G. (1992). Synthesis and antitumour activity of derivatives of curdlan and lichenan branched at C-6. Carbohydr. Res..

[80] Zhang Y., Tang W., Zheng Z., Nie G., Zhan Y., Mu X., Liu Y., Wang K. (2023). Metabolic degradation of polysaccharides from *Lentinus edodes* by Kupffer cells via the Dectin-1/Syk signaling pathway. Carbohydr. Polym..

[81] Kurczewska J. (2022). Recent reports on polysaccharide-based materials for drug delivery. Polymers.

[82] Zhang G., Wu J., Liu Y., Huang L., Qiao J., Liu X., Wei J., Guan Q. (2018). Effects of degree of substitution on stearic acid-modified *Bletilla striata* polysaccharides nanoparticles and interactions between nanoparticles and bovine serum albumin. Chin. Chem. Lett..

[83] Gref R., Minamitake Y., Peracchia M.T., Trubetskoy V., Torchilin V., Langer R. (1994). Biodegradable long-circulating polymeric nanospheres. Science.

[84] Kuttan R., Bhanumathy P., Nirmala K., George M. (1985). Potential anticancer activity of turmeric (*Curcuma longa*). Cancer Lett..

[85] Fang R.H., Kroll A.V., Gao W., Zhang L. (2018). Cell membrane coating nanotechnology. Adv. Mater..

[86] Li Y., Guo C., Chen Q., Su Y., Guo H., Liu R., Sun C., Mi S., Wang J., Chen D. (2022). Improvement of pneumonia by curcumin-loaded bionanosystems based on *platycodon grandiflorum* polysaccharides via calming cytokine storm. Int. J. Biol. Macromol..

[87] Gao J., Mao Y., Xiang C., Cao M., Ren G., Wang K., Ma X., Wu D., Xie H. (2021). Preparation of β-lactoglobulin/gum arabic complex nanoparticles for encapsulation and controlled release of EGCG in simulated gastrointestinal digestion model. Food Chem..

[88] Xiang C., Gao J., Ye H., Ren G., Ma X., Xie H., Fang S., Lei Q., Fang W. (2020). Development of ovalbumin-pectin nanocomplexes for vitamin D3 encapsulation: Enhanced storage stability and sustained release in simulated gastrointestinal digestion. Food Hydrocoll..

[89] Yoo H.S., Park T.G. (2004). Folate-receptor-targeted delivery of doxorubicin nano-aggregates stabilized by doxorubicin–PEG–folate conjugate. J. Control. Release.

[90] Zhang Y., Palanisamy S., Kwon M.-H., Ge Y., Kou F., Uthamapriya R.A., Lee D., Lee D.-J., Bao H., You S. (2025). A novel targeted anticancer drug delivery strategy: *Cnidium officinale* polysaccharide conjugated with carboxymethyl-5-fluorouracil and folic acid for ovarian cancer therapy. Int. J. Biol. Macromol..

[91] Fang L., Zhang W., Wang Z., Fan X., Cheng Z., Hou X., Chen D. (2019). Novel mitochondrial targeting charge-reversal polysaccharide hybrid shell/core nanoparticles for prolonged systemic circulation and antitumor drug delivery. Drug Deliv..

[92] Lv Q., Yang X., Wang M., Yang J., Qin Z., Kan Q., Zhang H., Wang Y., Wang D., He Z. (2018). Mitochondria-targeted prostate cancer therapy using a near-infrared fluorescence dye–monoamine oxidase A inhibitor conjugate. J. Control. Release.

[93] Wang Y., Zhang T., Hou C., Zu M., Lu Y., Ma X., Jia D., Xue P., Kang Y., Xu Z. (2019). Mitochondria-specific anticancer drug delivery based on reduction-activated polyprodrug for enhancing the therapeutic effect of breast cancer chemotherapy. ACS Appl. Mater. Interfaces.

[94] Dong L., Neuzil J. (2019). Targeting mitochondria as an anticancer strategy. Cancer Commun..

[95] Tan L., Wang Y., Ai G., Luo C., Chen H., Li C., Zeng H., Xie J., Chen J., Su Z. (2019). Dihydroberberine, a hydrogenated derivative of berberine firstly identified in *Phellodendri Chinese Cortex*, exerts anti-inflammatory effect via dual modulation of NF-κB and MAPK signaling pathways. Int. Immunopharmacol..

[96] Fang L., Lin H., Wu Z., Wang Z., Fan X., Cheng Z., Hou X., Chen D. (2020). In vitro/vivo evaluation of novel mitochondrial targeting charge-reversal polysaccharide-based antitumor nanoparticle. Carbohydr. Polym..

[97] Wu Q., Hu Y., Yu B., Hu H., Xu F.-J. (2023). Polysaccharide-based tumor microenvironment-responsive drug delivery systems for cancer therapy. J. Control. Release.

[98] Saikolappan S., Kumar B., Shishodia G., Koul S., Koul H.K. (2019). Reactive oxygen species and cancer: A complex interaction. Cancer Lett..

[99] Zhang Y., Li Y., Huang S., Zhang H., Lin Q., Gong T., Sun X., Zhang Z., Zhang L. (2021). Enhanced anti-metastatic therapy with down-regulation of heparinase expression by ROS-responsive micellar nanoparticles. Nanoscale.

[100] Gao N., Huang Y., Jing S., Zhang M., Liu E., Qiu L., Huang J., Muhitdinov B., Huang Y. (2024). Environment-responsive *dendrobium* polysaccharide hydrogel embedding manganese microsphere as a post-operative adjuvant to boost cascaded immune cycle against melanoma. Theranostics.

[101] Mendoza E., Ciriolo M., Ciccarone F. (2025). Hypoxia-induced reactive oxygen species: Their role in cancer resistance and emerging therapies to overcome it. Antioxidants.

[102] Szatrowski T.P., Nathan C.F. (1991). Production of large amounts of hydrogen peroxide by human tumor cells. Cancer Res..

[103] Fröhlich E. (2010). Proteases in cutaneous malignant melanoma: Relevance as biomarker and therapeutic target. Cell. Mol. Life Sci..

[104] Muniz-Bongers L.R., McClain C.B., Saxena M., Bongers G., Merad M., Bhardwaj N. (2021). MMP2 and TLRs modulate immune responses in the tumor microenvironment. JCI Insight.

[105] Han Q., Huang L., Wang Y., Sun S., Huang H., Li F., Wang F., Chen L., Zhang H., Wang Y. (2021). Platinum (II)-coordinated *Portulaca oleracea* polysaccharides as metal-drug based polymers for anticancer study. Colloids Surf. B Biointerfaces.

[106] Tao S., Song Y., Ding S., He R., Shi Q., Hu F. (2023). *Dendrobium officinale* polysaccharide-based carrier to enhance photodynamic immunotherapy. Carbohydr. Polym..

[107] Liu W., Jiang H., Xu J., Guo Y. (2025). Preparation and characterization of *Ganoderma lucidum* polysaccharide-based multifunctional tellurium nanorods to realize combination cancer therapy. Int. J. Biol. Macromol..

[108] Wang Y., Ma K., Kang M., Yan D., Niu N., Yan S., Sun P., Zhang L., Sun L., Wang D. (2024). A new era of cancer phototherapy: Mechanisms and applications. Chem. Soc. Rev..

[109] Yuan X., Zhou J.-L., Yuan L., Fan J., Yoon J., Zhang X.-B., Peng X., Tan W. (2025). Phototherapy: Progress, challenges, and opportunities. Sci. China Chem..

[110] Castano A.P., Mroz P., Hamblin M.R. (2006). Photodynamic therapy and anti-tumour immunity. Nat. Rev. Cancer.

[111] Yu W., Zhang Y., Yao L., Peng J., Tu Y., He B. (2024). Research progress on the prevention of tumor by fungal polysaccharides. Trends Food Sci. Technol..

